# Efficacy and safety of Xiaoer Feike Granules combined with azithromycin for Mycoplasma pneumoniae pneumonia: A systematic review and meta-analysis

**DOI:** 10.1097/MD.0000000000044287

**Published:** 2025-09-12

**Authors:** Xiaoqiong Xu, Gang Hu, Yunfeng Yu, Xinyu Yang, Keke Tong, Mengqing Wang, Fan Li

**Affiliations:** a Department of Respiratory, The 921st Hospital of the Joint Logistic Support Force of the Chinese People’s Liberation Army, Changsha, Hunan, China; b Department of Paediatrics, The First Hospital of Hunan University of Chinese Medicine, Changsha, Hunan, China; c School of Traditional Chinese Medicine, Hunan University of Chinese Medicine, Changsha, Hunan, China.

**Keywords:** azithromycin, meta analysis, Mycoplasma pneumoniae pneumonia, trial sequential analysis, Xiaoer Feike Granules

## Abstract

**Background:**

The application of Xiaoer Feike Granules (XFG) in respiratory diseases has garnered significant attention. This study aims to quantitatively assess the efficacy and safety of XFG when combined with azithromycin for the treatment of Mycoplasma pneumoniae pneumonia (MPP).

**Methods:**

A comprehensive search of 8 public databases was conducted for relevant clinical trials published before July 1, 2024. The meta-analysis and trial sequential analysis were performed on the included studies.

**Results:**

Compared with azithromycin alone, the combination of XFG and azithromycin significantly improved the clinical effective rate (RR = 1.17, 95% CI = 1.13–1.22, *P* < .00001), and reduced the duration of fever (MD = −1.67, 95% CI = −1.83 to −1.50, *P* < .00001), cough (MD = −1.85, 95% CI = −2.28 to −1.43, *P* < .00001), wheezing (MD = −2.07, 95% CI = −2.51 to −1.64, *P* < .00001), and rales (MD = −2.34, 95% CI = −3.11 to −1.57, *P* < .00001). It also lowered inflammatory markers, including C-reactive protein (MD = −8.22, 95% CI = −11.27 to −5.17, *P* < .00001), procalcitonin (MD = −0.50, 95% CI = −0.57 to −0.43, *P* < .00001), and tumor necrosis factor α (MD = −0.61, 95% CI = −0.72 to −0.49, *P* < .00001), while No significant difference was found in interleukin-6 levels (*P* = .11). In terms of safety, the combination of XFG and azithromycin significantly reduced the incidence of total adverse events (RR = 0.40, 95% CI = 0.19–0.84, *P* = .02), nausea and vomiting (RR = 0.42, 95% CI = 0.19–0.94, *P* = .03), abdominal pain and diarrhea (RR = 0.31, 95% CI = 0.13–0.75, *P* = .009), and erythra (RR = 0.27, 95% CI = 0.09–0.79, *P* = .02), with no significant differences in dizziness and headache (*P* = .24). Egger test indicated no evidence of publication bias in these outcomes (*P* > .05).

**Conclusion:**

Compared with azithromycin alone, XFG combined with azithromycin offers faster symptom resolution, reduced inflammation, and fewer adverse events. This combination may represent a safe and effective therapeutic strategy for MPP.

## 1. Introduction

Mycoplasma pneumoniae pneumonia (MPP) is a respiratory tract infection caused by Mycoplasma pneumoniae, primarily affecting preschool- and school-aged children.^[1]^ Epidemiological studies have shown that MPP accounts for approximately 40% of community-acquired pneumonia cases in children, with its incidence rising annually.^[2,3]^ If not promptly diagnosed and treated, pediatric MPP can lead to a range of extrapulmonary complications, including gastrointestinal disturbances, dermatological manifestations, cardiovascular symptoms, and neurological disorders.^[4,5]^ Currently, macrolide antibiotics, particularly azithromycin, are the first-line treatment for MPP in children.^[6]^ However, the widespread and prolonged use of azithromycin has led to a growing resistance of Mycoplasma pneumoniae, thereby diminishing its therapeutic efficacy.^[7]^ Moreover, azithromycin is associated with adverse effects such as nausea, vomiting, diarrhea, dizziness, tinnitus, leukopenia, and rash, raising concerns among both clinicians and caregivers.^[8]^ These issues underscore the urgent need for safe and effective adjunctive therapies for MPP.

Xiaoer Feike Granules (XFG) is a traditional Chinese medicine formulation developed by Chinese researchers. It is composed of multiple herbal and animal-derived ingredients, including *Panax ginseng* C.A. Mey [Araliaceae; Panax ginseng radix et rhizoma], *Poria cocos* F.A. Wolf [Polyporaceae; Poria cocos sclerotium], *Atractylodes macrocephala* Koidz [Asteraceae; Atractylodes macrocephala rhizoma], *Citrus reticulata* Blanco [Rutaceae; Citrus reticulata pericarpium], *Astragalus mongholicus* Bunge [Fabaceae; Astragalus mongholicus radix], *Glehnia littoralis* (A. Gray) F. Schmidt ex Miq [Apiaceae; Glehnia littoralis radix], *Lycium barbarum* L. [Solanaceae; Lycium barbarum fructu], *Ophiopogon japonicus* (Thunb.) Ker Gawl [Asparagaceae; Ophiopogon japonicus radix], *Cinnamomum aromaticum* Nees [Lauraceae; Cinnamomum aromaticum ramulus], *Zingiber officinale* Roscoe [Zingiberaceae, Zingiber officinale rhizoma], *Morus alba* L. [Moraceae; Morus alba cortex], *Trichosanthes kirilowii* Maxim [Cucurbitaceae; Trichosanthes kirilowii fructus], *Tussilago farfara* L. [Asteraceae; Tussilago farfara flos], *Aster tataricus* L.F. [Asteraceae; Aster tataricus radix et rhizoma], *Gallus gallus domesticus* Brisson [Phasianidae; Gallus gallus domesticus corneum], *Arisaema erubescens* (Wall.) Schott [Araceae; Arisaema erubescens tuber], *Trionyx sinensis* Wiegmann [Trionychidae; Trionyx sinensis carapax], *Artemisia annua* L. [Asteraceae; Artemisia annua herba], *Rheum palmatum* L. [Polygonaceae; Rheum palmatum radix et rhizoma], *Glycyrrhiza uralensis* Fisch [Polygonaceae; Glycyrrhiza uralensis radix et rhizoma]. XFG has shown anti-inflammatory and immunomodulatory effects in clinical research.^[9,10]^ Subsequent studies have suggested that XFG may relieve common symptoms of MPP, such as fever, cough, and wheezing, and may also shorten the duration of hospitalization.^[11,12]^ However, due to the limited availability of high-quality clinical evidence, its overall benefits and potential risks remain unclear. Therefore, this study aims to conduct a systematic review and meta-analysis to quantitatively assess the efficacy and safety of XFG combined with azithromycin for MPP. The findings aim to provide objective and reliable evidence to support its clinical application.

## 2. Materials and methods

This study was conducted in accordance with the Preferred Reporting Items for Systematic Reviews and Meta-Analyses (PRISMA) guidelines.^[13]^ Since the data were obtained from publicly available databases, no additional ethical review or informed consent was required for this study.

### 2.1. Literature search

A comprehensive literature search was conducted across 8 public databases using a combination of subject terms and supplementary keywords. The subject terms included Xiaoer Feike Granules, azithromycin, and MPP. Additional keywords were sourced from MeSH and SinoMed. The databases searched were China National Knowledge Infrastructure, SinoMed, Wanfang Data, China Science and Technology Journal Database, Embase, PubMed, the Cochrane Library, and Web of Science. The search covered all publications from the inception of each database to July 1, 2024, with no restrictions on language or publication type.

### 2.2. Inclusion and exclusion criteria

Inclusion criteria: Participants: Patients meeting the diagnostic criteria for MPP.^[14]^ Intervention: The intervention group received XFG in combination with azithromycin. Control: The control group received azithromycin alone. Outcomes: Efficacy outcomes included clinical effective rate, symptom resolution times (fever, cough, wheezing, and rales), and inflammatory biomarkers (C-reactive protein [CRP], procalcitonin [PCT], tumor necrosis factor-α [TNF-α], and interleukin-6 [IL-6]). The primary efficacy outcome was the clinical effective rate, defined as the proportion of patients showing symptom improvement or resolution. Safety outcomes included the incidence of total and specific adverse events. Study design: Randomized controlled trials (RCTs).

Exclusion criteria: Reviews, animal studies, and case reports. Duplicate publications. Studies with incomplete or unavailable data.

### 2.3. Literature screening, data extraction, and risk assessment of bias

All retrieved records were imported into EndNote X9 for reference management. Two reviewers independently screened the titles, abstracts, and full texts according to the inclusion and exclusion criteria to determine eligible studies. Discrepancies were resolved through discussion or consultation with a third reviewer. From the included studies, the following data were extracted: first author, year of publication, sample size, mean age, gender ratio, intervention details, and treatment duration. According to the Cochrane Handbook for Systematic Reviews of Interventions, the Risk of Bias (ROB)-1 tool was used to assess publication bias in the included studies. These tasks were performed independently by 2 reviewers, with disagreements resolved by a third party.

### 2.4. Statistical analysis

Meta-analysis was performed using RevMan 5.3 (The Cochrane Collaboration, Copenhagen, Denmark). Heterogeneity was assessed using the *I*^2^ statistic. If *I*^2^ < 50%, a fixed-effect model was used; if *I*^2^ ≥ 50%, a random-effects model was applied. The risk ratio (RR) with 95% confidence intervals (CI) was calculated for dichotomous outcomes, and mean difference with 95% CI were calculated for continuous outcomes. The *P* < .05 was considered statistically significant. Trial sequential analysis (TSA) was performed using TSA version 0.9.5.10. Type I and type II error rates were set at 5% and 20%, respectively. The results of meta-analysis were considered conclusive if the cumulative *Z*-curve crossed the monitoring boundaries. Subsequently, sensitivity analysis was conducted using a leave-one-out approach. If the overall results remained stable after omitting each study in turn, the findings were considered robust. Additionally, subgroup analysis was conducted to assess the influence of factors such as disease duration and treatment duration on the clinical effective rate. Finally, Egger test was performed using Stata 15.0 (StataCorp LLC, College Station ) to evaluate potential publication bias. The *P* > .05 was considered indicative of no significant publication bias.

## 3. Results

### 3.1. Literature screening

**Figure 1.**
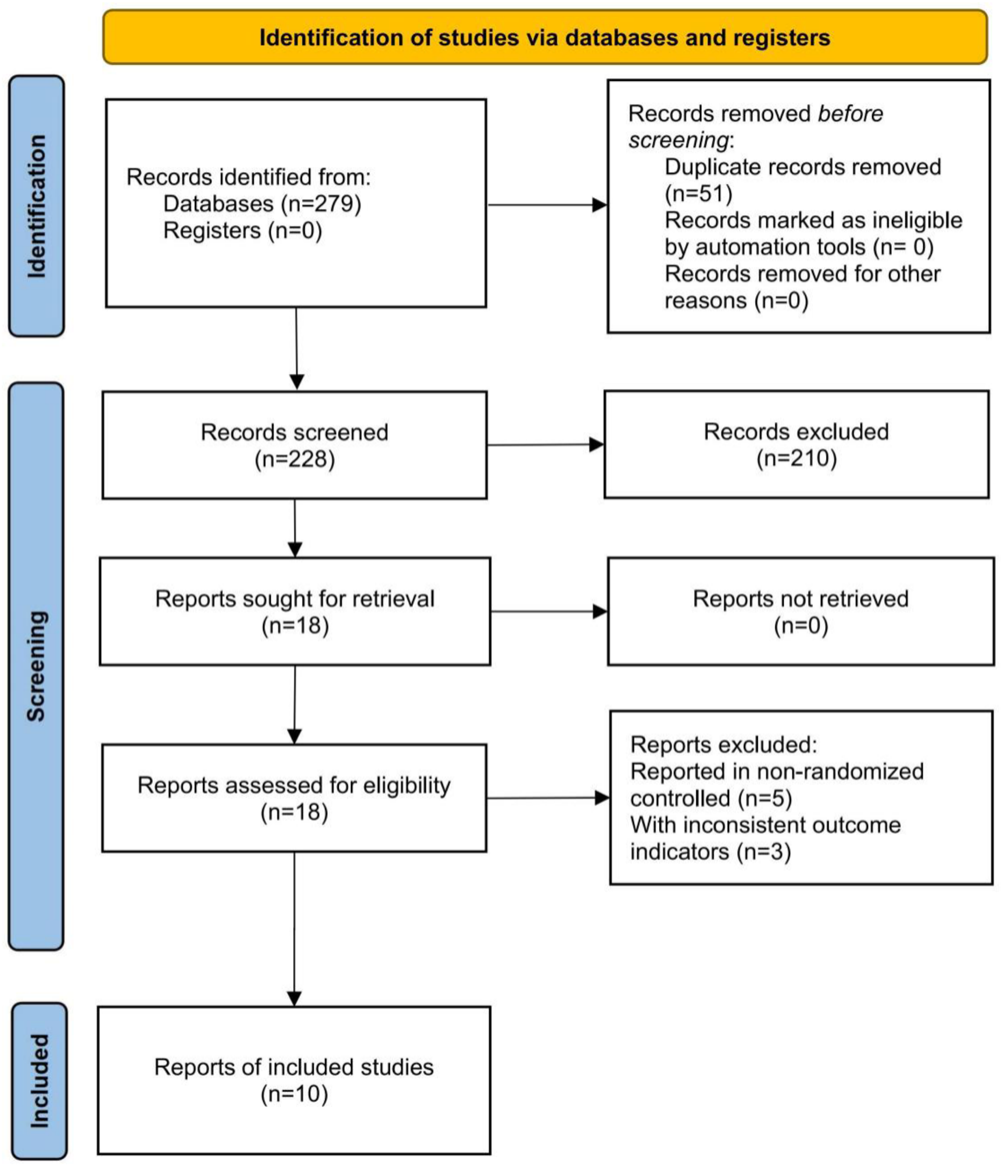
Literature screening process diagram.

A total of 279 records were identified through the literature search. After removing 51 duplicates, 218 articles were excluded based on titles, abstracts, or full texts for not meeting the eligibility criteria. Ultimately, 10 articles were included in the analysis^[15–24]^ (Fig. 1).

### 3.2. Basic characteristics of the included studies

The 10 included studies involved a total of 1210 patients, of whom 606 received XFG combined with azithromycin, and 604 received azithromycin alone. All studies were conducted in China and published between 2018 and 2022 (Table 1). Details regarding the source and quality control of XFG used in each study are presented in Table S1 (Supplemental Digital Content, https://links.lww.com/MD/P907).

**Table 1:** Basic characteristics of the included studies.

Author name	Sample size	Age (yr)	Male (%)	Disease duration (d)	Azithromycin	XFG	Treatment duration (d)
Duan CH, 2019^[15]^	103/103	5.3	51.9	/	10 mg/(kg·d)	1–4 yr: 9 g/d	3
						5–10 yr: 18 g/d	
Liao Z, 2018^[16]^	53/53	4.4	52.8	/	10 mg/(kg·d)	<1 yr: 6 g/d	14
						1–4 yr: 9 g/d	
						5–8 yr: 18 g/d	
Lin HQ, 2020^[17]^	60/60	6.7	51.7	8.2	500 mg/d	2–4 yr: 9 g/d	7
						>5 yr: 18 g/d	
Ma YX, 2020^[18]^	67/65	7.6	53.8	8.2	10 mg/(kg·d)	12 g/d	14
Mei H, 2022^[19]^	40/40	8.1	56.3	/	10 mg/(kg·d)	1–4 yr: 9 g/d	14
						5–12 yr: 18 g/d	
Song B, 2019^[20]^	60/60	8.4	52.5	/	10 mg/(kg·d)	<1 yr: 6 g/d	7
						1–4 yr: 9 g/d	
						>5 yr: 18 g/d	
Wang CL, 2021^[21]^	41/41	7.5	57.3	5.0	10 mg/(kg·d)	18 g/d	14
Yang YQ, 2021^[22]^	47/47	8.3	55.3	8.5	10 mg/(kg·d)	<1 yr: 6 g/d	7
						1–4 yr: 9 g/d	
						>4 yr: 18 g/d	
Zhang JM, 2020^[23]^	75/75	3.7	51.3	/	<15 kg: 100 mg/d	3 g/d	14
					15~25 kg: 200 mg/d		
					26–35 kg: 300 mg/d		
Zou QM, 2021^[24]^	60/60	4.3	60.3	2.8	10 mg/(kg·d)	1–4 yr: 9 g/d	14
						5–8 yr: 18 g/d	

Baseline information such as gender, age, and disease duration were comparable between the experimental and control groups in each included study.

XFG = Xiaoer Feike Granules.

### 3.3. ROB assessment

**Figure 2.**
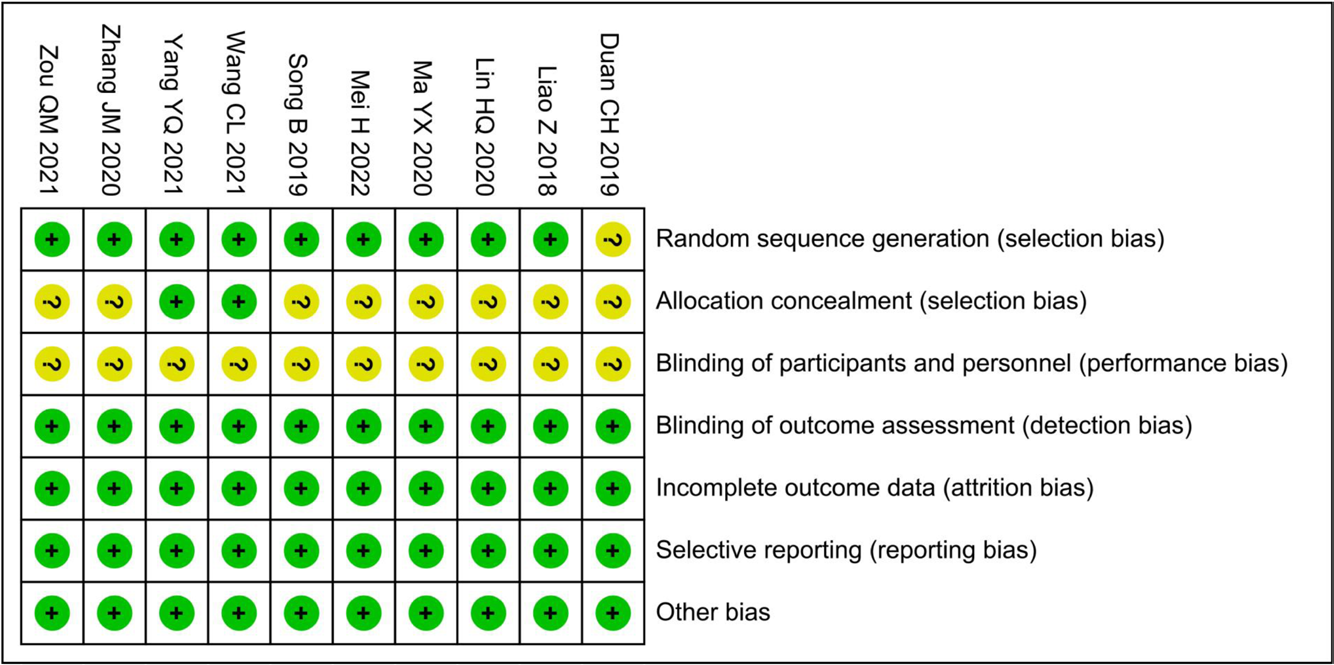
Risk of bias graph.

Among the 10 studies, 1 did not clearly report its randomization method. Eight studies lacked explicit descriptions of allocation concealment, and none provided clear information on whether participant blinding was implemented. Therefore, these domains were deemed to have an unclear ROB. However, the ROB was assessed as low in other domains (Fig. 2).

### 3.4. Meta-analysis

#### 3.4.1. Clinical effective rate

**Figure 3.**
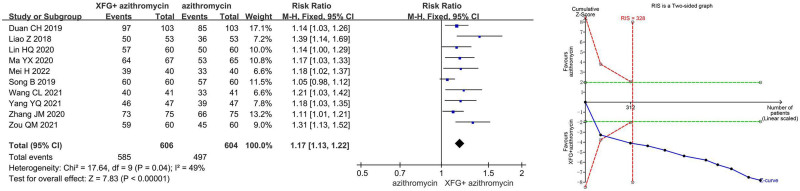
The meta-analysis and trial sequential analysis of the clinical effective rate.

Meta-analysis showed that compared to azithromycin alone, XFG combined with azithromycin significantly improved the clinical effective rate by 17% (RR = 1.17, 95% CI = 1.13–1.22, *P* < .00001). TSA confirmed the conclusiveness of this result (Fig. 3).

#### 3.4.2. Symptom resolution time

**Figure 4.**
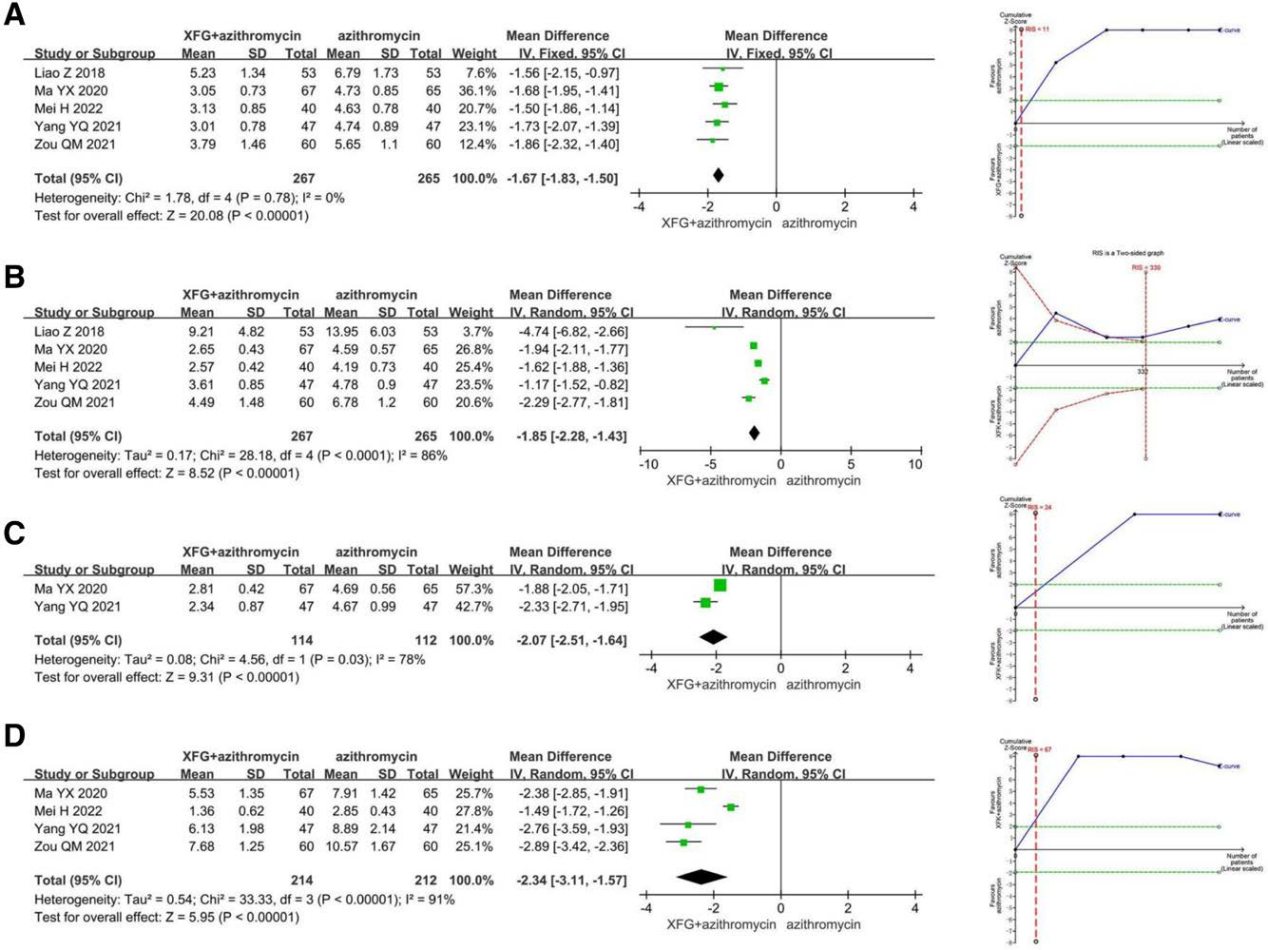
The meta-analysis and trial sequential analysis of symptom resolution time: (A) fever; (B) cough; (C) wheeze; (D) rale.

Compared to azithromycin, XFG combined with azithromycin significantly reduced the resolution times of fever by 1.67 days (MD = −1.67, 95% CI = −1.83 to −1.50, *P* < .00001), cough by 1.85 days (MD = −1.85, 95% CI = −2.28 to −1.43, *P* < .00001), wheeze by 2.07 days (MD = −2.07, 95% CI = −2.51 to −1.64, *P* < .00001), and rale by 2.34 days (MD = −2.34, 95% CI = −3.11 to −1.57, *P* < .00001). TSA confirmed that all of these findings were conclusive (Fig. 4).

#### 3.4.3. Inflammatory markers

**Figure 5.**
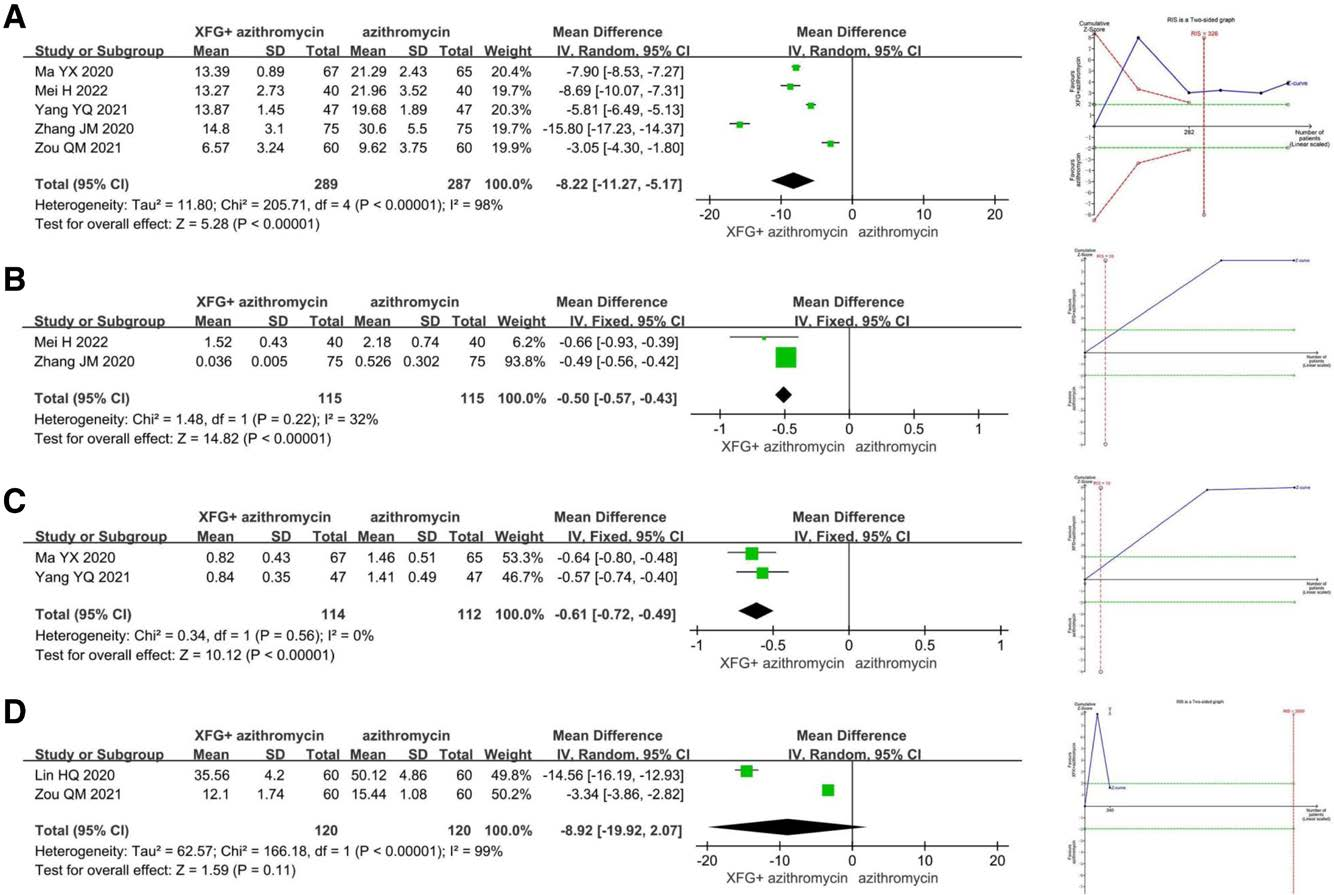
The meta-analysis and trial sequential analysis of inflammatory markers: (A) C-reactive protein (CRP); (B) procalcitonin (PCT); (C) tumor necrosis factor-alpha (TNF-α); (D) interleukin-6 (IL-6).

Compared with azithromycin alone, XFG combined with azithromycin significantly reduced CRP by 8.22 mg/L (MD = −8.22, 95% CI = −11.27 to −5.17, *P* < .00001), PCT by 0.50 ng/mL (MD = −0.50, 95% CI = −0.57 to −0.43, *P* < .00001), and TNF-α levels by 0.61 mg/L (MD = −0.61, 95% CI = −0.72 to −0.49, *P* < .00001), while had no significant effect on IL-6 levels (MD = −8.92, 95% CI = −19.92–2.07, *P* = .11). TSA confirmed that the results for CRP, PCT, and TNF-α were conclusive (Fig. 5).

#### 3.4.4. Adverse events

Meta-analysis showed that the incidence of adverse events was significantly lower in the combination group: Total adverse events by 60% (RR = 0.40, 95% CI = 0.19–0.84, *P* = .02), nausea and vomiting decreased by 58% (RR = 0.42, 95% CI = 0.19–0.94, *P* = .03), abdominal pain and diarrhea decreased by 69% (RR = 0.31, 95% CI = 0.13–0.75, *P* = .009), and erythra decreased by 73% (RR = 0.27, 95% CI = 0.09–0.79, *P* = .02). However, there was no significant difference in the incidence of dizziness and headache (RR = 0.33, 95% CI = 0.05–2.06, *P* = .24). TSA supported the conclusiveness of the findings for total adverse events, abdominal pain and diarrhea, and erythra (Table 2).

**Table 2:** The meta-analysis and trial sequential analysis for safety outcomes.

Outcome	Experimental group	Control group	*I* ^2^	RR (95% CI)	*P*-value	TSA
Total adverse events	29/439	79/437	62	0.40 (0.19–0.84)	.02	Yes
Nausea and vomiting	8/271	19/269	10	0.42 (0.19–0.94)	.03	No
Dizziness and headache	1/107	4/105	0	0.33 (0.05–2.06)	.24	No
Abdominal pain and diarrhea	6/311	20/309	10	0.31 (0.13–0.75)	.009	Yes
Erythra	4/211	15/209	10	0.27 (0.09–0.79)	.02	Yes

### 3.5. Sensitivity analysis

Sensitivity analysis using the leave-one-out method demonstrated robust results for the following outcomes: clinical effective rate, resolution times of fever, cough, wheezing, and rales, as well as CRP, PCT, TNF-α, total adverse events, and dizziness and headache. However, the results for IL-6, nausea and vomiting, abdominal pain and diarrhea, and erythra lacked robustness.

### 3.6. Subgroup analysis

Subgroup analyses were performed to explore the impact of clinical factors on the primary outcome. When stratified by disease duration, patients with MPP of ≤5 days showed a significant benefit from the combination of XFG and azithromycin (RR = 1.27, 95% CI = 1.14–1.42, *P* < .0001), as did those with disease duration ≥ 8 days (RR = 1.16, 95% CI = 1.08–1.25, *P* < .0001), indicating that the combination therapy was effective across different stages of illness. In terms of treatment duration, both shorter (≤7 days) and longer (>7 days) courses of XFG combined with azithromycin significantly improved the clinical effective rate. Specifically, the clinical effective rate improved significantly with both shorter (RR = 1.13, 95% CI = 1.07–1.19, *P* < .0001) and longer (RR = 1.21, 95% CI = 1.15–1.29, *P* < .00001) treatment duration (Table 3). These findings suggest that the therapeutic effect of XFG is consistent regardless of disease or treatment duration.

**Table 3:** Subgroup analysis based on disease and treatment duration.

Subject	Subgroup	*I* ^2^	RR (95% CI)	*P*-value
Disease duration	≤5 d	0	1.27 (1.14–1.42)	<.0001
	≥8 d	0	1.16 (1.08–1.25)	<.0001
Treatment duration	≤7 d	34	1.13 (1.07–1.19)	<.0001
	>7 d	31	1.21 (1.15–1.29)	<.00001

### 3.7. Publication bias

**Figure 6.**
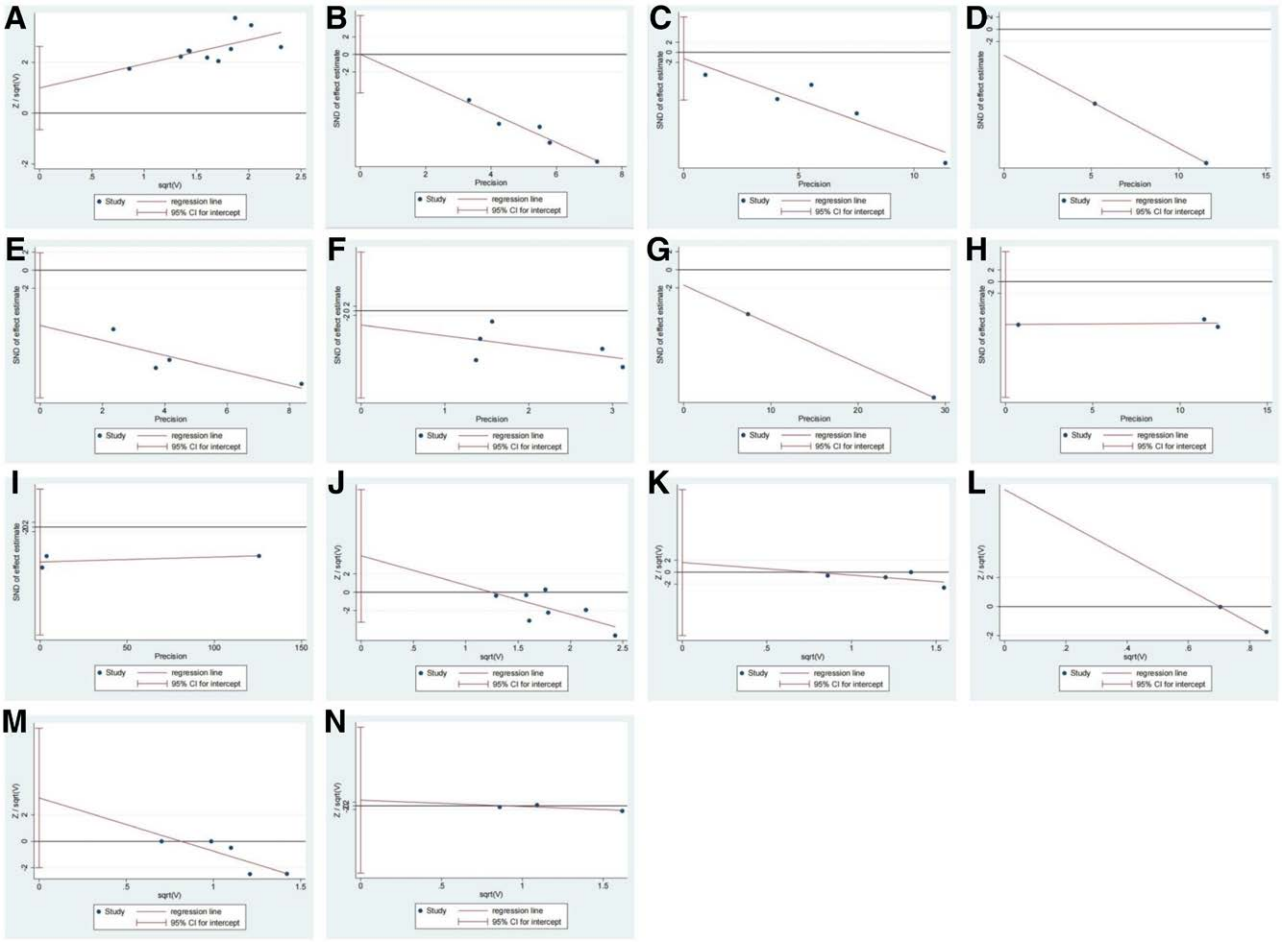
Egger test for publication bias: (A) clinical effective rate; (B) resolution time of fever; (C) resolution time of cough; (D) resolution time of wheeze; (E) resolution time of rale; (F) C-reactive protein (CRP); (G) procalcitonin (PCT); (H) tumor necrosis factor-alpha (TNF-α); (I) interleukin-6 (IL-6); (J) total adverse events; (K) nausea and vomiting; (L) dizziness and headache; (M) abdominal pain and diarrhea; (N) erythra.

Egger test showed no evidence of publication bias for all outcomes, including clinical effective rate (*P* = .202), resolution time of fever (*P* = .991), resolution time of cough (*P* = .666), resolution time of wheezing (*P* = 1.000), resolution time of rales (*P* = .082), CRP (*P* = .577), PCT (*P* = 1.000), TNF-α (*P* = .085), IL-6 (*P* = .103), total adverse events (*P* = .218), nausea and vomiting (*P* = .625), dizziness and headache (*P* = 1.000), abdominal pain and diarrhea (*P* = .143), and erythra (*P* = .498), as shown in Figure 6.

## 4. Discussion

Mycoplasma pneumoniae is a common pathogen responsible for community-acquired pneumonia in children.^[25]^ Infected children typically present with persistent fever, cough, and wheezing, and in severe cases, the condition can become life-threatening.^[26,27]^ However, the widespread use of azithromycin has led to a notable increase in Mycoplasma pneumoniae resistance, resulting in reduced treatment efficacy.^[28]^ This underscores the urgent need for safe and effective adjunctive therapies for managing MPP. XFG, a traditional Chinese medicine commonly used to treat pediatric cough, has demonstrated the potential to enhance peripheral blood T cell subsets and suppress inflammatory responses,^[29]^ indicating possible therapeutic benefits in MPP. Nevertheless, the absence of high-quality evidence has hindered a clear understanding of the efficacy and safety of XFG when used in combination with azithromycin for MPP. To address this gap, we conducted the first meta-analysis and TSA in this context, incorporating 10 clinical trials involving 1210 patients, with the aim of providing evidence-based guidance for clinical application of XFG.

In terms of clinical symptoms and signs, the meta-analysis revealed that the combination of XFG and azithromycin significantly increased the clinical effective rate by 17%, and shortened the resolution time of fever by 1.67 days, cough by 1.85 days, wheeze by 2.07 days, and rale by 2.34 days compared with azithromycin alone. These findings were confirmed to be conclusive by TSA. This suggests that XFG facilitates the relief of clinical symptoms, thereby reducing disease burden and accelerating recovery. Supporting this, Mei et al^[19]^ reported that XFG significantly reduced hospital stay duration in children with MPP by promoting symptom resolution, ultimately easing the financial burden on families.

At the inflammatory level, XFG combined with azithromycin significantly reduced levels of CRP by 8.22 mg/L, PCT by 0.50 ng/mL, and TNF-α by 0.61 mg/L, compared to azithromycin alone. These results were also deemed conclusive. TNF-α plays a key role in mediating pulmonary inflammation and exacerbating lung injury.^[30]^ CRP, an acute-phase protein produced by the liver, is a vital biomarker for diagnosing pediatric pneumonia.^[31]^ PCT serves as a specific indicator for bacterial infection, with elevated levels indicating more severe Mycoplasma pneumoniae infection.^[32]^ Therefore, the observed reductions suggest that XFG may alleviate pulmonary inflammatory damage, potentially through inhibitory effects on Mycoplasma pneumoniae. Interestingly, although both included studies indicated a beneficial effect of XFG on reducing IL-6 levels, the overall meta-analysis produced a nonsignificant result. Sensitivity analysis showed that this negative outcome was not robust and was highly sensitive to the statistical model used. When a fixed-effects model was applied, the result became significant (MD = −4.38, 95% CI = −4.87 to −3.88, *P* < .00001). As the included studies showed no obvious clinical or methodological heterogeneity, this suggests the presence of statistical rather than true heterogeneity. We therefore speculate that XFG may indeed reduce IL-6 levels and exert anti-inflammatory effects. Further studies are warranted to clarify this effect and avoid overestimation of negative outcomes due to model choice.

Subgroup analysis demonstrated that XFG improved clinical effectiveness in patients with both early-stage (≤ 5 days) and later-stage (≥ 8 days) disease durations, highlighting its efficacy across different phases of MPP. Additionally, both short-term (≤ 7 days) and longer-term (> 7 days) treatment durations resulted in clinical benefits. These findings suggest that the therapeutic effect of XFG is consistent regardless of disease duration or treatment course, allowing clinicians to tailor XFG treatment according to individual patient needs.

With regard to safety, meta-analysis showed that the combination of XFG and azithromycin significantly reduced the incidence of total adverse events by 60% compared to azithromycin alone. This result was also confirmed by TSA. Notably, azithromycin is often associated with gastrointestinal side effects such as nausea, vomiting, and diarrhea, as well as skin rashes, which are the main reasons for treatment discontinuation.^[8]^ The present analysis showed that co-administration with XFG reduced the incidence of nausea and vomiting by 58%, abdominal pain and diarrhea by 69%, and erythema by 73%. The incidence of dizziness and headache was comparable between the 2 groups, suggesting that the addition of XFG does not increase neurological risks. These results suggest that XFG may alleviate azithromycin-related adverse effects, potentially improving pediatric patient compliance. However, it is important to note that these findings pertain only to the combination therapy and do not reflect the safety profile of XFG when used alone. In a phase II clinical trial involving 193 children with acute bronchiolitis, no drug-related adverse events were reported with XFG,^[33]^ supporting its good safety profile. Nevertheless, due to the limited robustness of some safety endpoints beyond total adverse events, further large-scale, multicenter RCTs are needed to confirm these safety findings.

Despite strict adherence to guidelines, this study has several limitations that should be acknowledged. First, the included trials did not report detailed information regarding the implementation of blinding methods, which may introduce methodological heterogeneity and increase the risk of performance bias. Second, the diagnostic criteria used in the studies varied, including the 2008 TCM Diagnosis and Treatment Guidelines for Pediatric Pneumonia Wheezing,^[34]^ the 2012 TCM Diagnosis and Treatment Guidelines for Pediatric Diseases,^[35]^ and the 2015 Chinese Medical Association Respiratory Group Standards for Pediatric Respiratory Diseases.^[14]^ These discrepancies may contribute to clinical heterogeneity. Third, due to the limited number and sample size of the included studies, the meta-analysis results for IL-6, nausea and vomiting, abdominal pain and diarrhea, and erythema lack robustness, which may reduce the reliability of these specific outcomes. Fourth, as XFG is primarily marketed in China, all included studies were conducted within Chinese populations. This geographical and ethnic homogeneity limits the generalizability of the findings to other populations, particularly those outside East and Southeast Asia. Fifth, although this study confirmed the effectiveness of XFG in treating MPP, the available data were insufficient to assess the specific efficacy and safety of XFG combined with azithromycin across different pediatric age groups.

Given these limitations, several improvements are recommended for future research. First, researchers should design and conduct multicenter, large-scale, double-blind RCTs, with particular attention to evaluating changes in inflammatory markers. Such studies would enhance the quality of evidence supporting the use of XFG in MPP. Second, due to the low frequency of adverse events and the need for adequate statistical power, larger patient cohorts should be observed to thoroughly assess the safety profile of XFG. Third, age-stratified analyses should be conducted to explore the differential efficacy and safety of XFG in various pediatric age groups, while adequately controlling for confounding factors. Finally, it is important to establish clinical research centers in countries across Europe, the Americas, and Africa. This would allow for the investigation of the therapeutic effects of XFG combined with azithromycin in racially and ethnically diverse populations, thereby improving the generalizability and global applicability of the findings.

## 5. Conclusion

The combination of XFG and azithromycin significantly improves clinical efficacy, accelerates symptom resolution, mitigates inflammatory responses, and reduces the incidence of overall and gastrointestinal adverse events in patients with MPP. These findings suggest that XFG may be a promising adjunctive therapy that warrants consideration in the clinical management of MPP.

## Acknowledgments

We would like to thank Dr Fei Zhang from Hunan University of Chinese Medicine for his valuable assistance in reviewing the statistical analyses.

## Author contributions

**Conceptualization:** Xiaoqiong Xu, Gang Hu, Yunfeng Yu.

**Data curation:** Xinyu Yang, Keke Tong.

**Formal analysis:** Xinyu Yang, Keke Tong.

**Methodology:** Gang Hu, Yunfeng Yu.

**Supervision:** Mengqing Wang, Fan Li.

**Writing – original draft:** Gang Hu, Yunfeng Yu, Xinyu Yang, Keke Tong.

**Writing – review & editing:** Xiaoqiong Xu, Mengqing Wang, Fan Li.

## Supplementary Material


